# An Assessment of Myotube Morphology, Matrix Deformation, and Myogenic mRNA Expression in Custom-Built and Commercially Available Engineered Muscle Chamber Configurations

**DOI:** 10.3389/fphys.2018.00483

**Published:** 2018-05-08

**Authors:** Julia M. Jones, Darren J. Player, Neil R. W. Martin, Andrew J. Capel, Mark P. Lewis, Vivek Mudera

**Affiliations:** ^1^Division of Surgery and Interventional Science, Institute of Orthopaedics and Musculoskeletal Science, University College London, London, United Kingdom; ^2^School of Sport, Exercise and Health Sciences, Loughborough University, Loughborough, United Kingdom

**Keywords:** skeletal muscle, tissue engineering, C2C12, myotubes, commercially available muscle chamber, custom-built muscle chamber

## Abstract

There are several three-dimensional (3D) skeletal muscle (SkM) tissue engineered models reported in the literature. 3D SkM tissue engineering (TE) aims to recapitulate the structure and function of native (*in vivo*) tissue, within an *in vitro* environment. This requires the differentiation of myoblasts into aligned multinucleated myotubes surrounded by a biologically representative extracellular matrix (ECM). In the present work, a new commercially available 3D SkM TE culture chamber manufactured from polyether ether ketone (PEEK) that facilitates suitable development of these myotubes is presented. To assess the outcomes of the myotubes within these constructs, morphological, gene expression, and ECM remodeling parameters were compared against a previously published custom-built model. No significant differences were observed in the morphological and gene expression measures between the newly introduced and the established construct configuration, suggesting biological reproducibility irrespective of manufacturing process. However, TE SkM fabricated using the commercially available PEEK chambers displayed reduced variability in both construct attachment and matrix deformation, likely due to increased reproducibility within the manufacturing process. The mechanical differences between systems may also have contributed to such differences, however, investigation of these variables was beyond the scope of the investigation. Though more expensive than the custom-built models, these PEEK chambers are also suitable for multiple use after autoclaving. As such this would support its use over the previously published handmade culture chamber system, particularly when seeking to develop higher-throughput systems or when experimental cost is not a factor.

## Introduction

Tissue engineered (TE) three-dimensional (3D) constructs allow for complex representation of several tissue types *in vitro*, including skeletal muscle (SkM). The goal of 3D SkM TE is to emulate native muscle (Ostrovidov et al., 2014; Perniconi and Coletti, 2014; Khodabukus and Baar, 2015), which includes the geometrical, topographical, and physical features of the targeted tissue (Mohanty et al., 2015). Native SkM tissue consists of parallel arrays of multinucleated myofibers of relatively equal size (Bian and Bursac, 2008; Riboldi et al., 2008; Gillies and Lieber, 2011) that are surrounded by an extracellular matrix (ECM; Gillies and Lieber, 2011). Therefore, a requirement of SkM TE is the development of uniaxially aligned myotubes, typically achieved utilizing a high cell density of muscle precursor cells, seeded on or within a scaffold anchored between two secured fixed points (Sakar et al., 2012).

Several published TE 3D SkM models have been reported in the literature, for example, cylindrical (Okano and Matsuda, 1998; Vandenburgh et al., 2008), mandrel/cylindrical (Okano and Matsuda, 1997), tubular (Huang et al., 2005; Khodabukus and Baar, 2009; Martin et al., 2015), and rectangular/cuboidal systems have been previously demonstrated (Eastwood et al., 1996; Cheema et al., 2003, 2005; Mudera et al., 2010; Smith et al., 2012; Hodgson, 2015; key features summarized in **Table 1**). Whilst these models (regardless of geometry or size) represent simplified versions of the desired complex tissue, 3D SkM constructs have been shown to be capable of supporting cell growth and maturation (Cen et al., 2008; Bian and Bursac, 2009), force production (Powell et al., 2002; Ito et al., 2014), and supporting co-culture with organotypic cell types such as motor neurons (Morimoto et al., 2013; Martin et al., 2015; Smith et al., 2016).

**Table 1:** A comparison of commonly published skeletal muscle models.

Publication features	Vandenburgh et al., 1996	Okano and Matsuda, 1998	Vandenburgh et al., 2008	Chiron et al., 2012	Smith et al., 2012	Martin et al., 2015
**Features of published skeletal muscle chambers**					
Attachment/fixed points	Velcro and stainless-steel pins	Fixed points	Flexible silicone posts	Silicone pins	Polyethylene mesh	Stainless-steel pins
Construct volume	400 μL	n/a	100 μL	150 μL	3200 μL	700 μL
Matrix	Collagen and matrigel^®^	Collagen	Collagen, atrigel^®^ and fibrin	Fibrin	Collagen	Thrombin and fibrin

**Matrices type**			**Natural**			

Seeded cell type	C2C12 mouse myoblasts	C2C12 mouse myoblasts	Primary mouse myoblasts	Primary HDMCs	Primary rat DMCs	C2C12 mouse myoblasts or primary human DMCs
Geometric configuration of chamber	Rectangular	Mandrel – ring shaped	Cylindrical	Rectangular	Rectangular	Circular – then rolled cylindrical tubes
Chamber design type	Custom-built	Custom-built	Manufactured	Custom-built	Custom-built	Custom-built
Chamber/well type	Commercially available silicone tubing	Agarose gel ring	Custom-built wells with posts	Custom-built	Commercially available singular rectangular wells	Custom-built wells with pins


*The chambers used for 3D TE have three main corresponding features. (1) They are anchored by fixed point(s). (2) They have a geometric configuration that is rectangular. (3) The chambers are used with a natural (compliant) matrix. The hydrogel matrix/scaffold used for supporting cells varies between the models, as does construct volume, cell type, and seeding density per milliliter (latter not displayed).*

**FIGURE 1:**
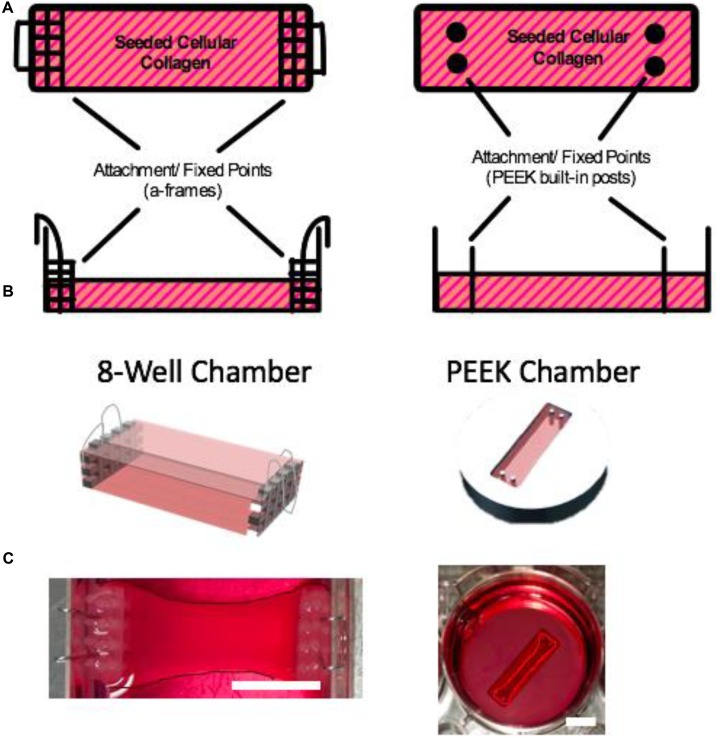
Muscle chambers. **(A)** Schematic illustration of the muscle chambers with their respective attachment points. **(B)** CAD model of the respective chambers (images **A** and **B** are not to scale). **(C)** The 8WC and PEEK chambers used in our experimental research. The images have been enhanced by outlining the constructs to define their contrast within their chamber against the surrounding medium. Scale bars = 10 mm.

These 3D TE SkM models are typically cultured within chambers, which are fabricated using bespoke techniques and/or custom designs. As such, a large number of models exist (Vandenburgh et al., 1996, 2008; Dennis and Kosnik, 2000; Powell et al., 2002; Cheema et al., 2003, 2005; Chiron et al., 2012; Snyman et al., 2013; to list a few, summarized in **Table 1**). Thus, manufacturing reproducible chambers can be problematic, often resulting in experimental variation. In the present study, TE SkM constructs were fabricated in a precision manufactured and commercially available polyether ether ketone (PEEK) 3D SkM TE culture chamber, and were compared to an 8-well 3D SkM TE culture chamber (8WC) model based on the attachment of the constructs to handmade “floatation bars” (Smith et al., 2012). Both SkM configurations have a similar rectangular geometry, but have variations in cellular volumes, chamber design, as well as the modes of attachment for the SkM construct (**Figure 1**). These variations enable the investigation of myotube parameters in contrasting systems, similar to those presented in the literature. Furthermore, by utilizing a precision manufactured and commercially available system such as the proposed PEEK chamber, it is foreseen that repeatability and replication of SkM constructs should be improved above the custom-built platform. Moreover, adopting an approach utilizing precision manufacturing would also facilitate movement toward scalable, higher-throughput systems. When seeking to evaluate the success of a given model, it is important to consider a number of myogenic parameters. Thus, it is necessary to examine the expression of key myogenic genes and the morphological characteristics of the seeded cells to assess the extent of differentiation. To this end, the purpose of this study was to understand the differences in basic myotube, myogenic mRNA and ECM characteristics when C2C12 myoblasts were cultured in a previously published custom handmade system (8WC) compared with a precision manufactured configuration (PEEK).

## Materials and Methods

### Cell Culture

C2C12 myoblasts (Public Health England sourced from ATCC) at passages 3–12 were maintained in basal Dulbecco’s Modified Eagle’s Medium (DMEM) (Sigma-Aldrich, United Kingdom) supplemented with 20% v/v fetal calf serum [First Link (UK) Ltd., United Kingdom] and 1% v/v penicillin–streptomycin (Gibco Life Technologies, United Kingdom). All cell cultures were kept in a humidified incubator at 37°C and 5% CO_2_ for the duration of the experiment.

### Chamber Configurations

Constructs cultured in the 8WC (**Figure 1**) are setup in commercially available treated tissue culture plates (Nunc^TM^, Thermo Fisher Scientific, United Kingdom), with the addition of polydimethylsiloxane (PDMS, Dow Corning, United States) walls to divide the chamber to the required dimensions for the polymerization process. The construct’s attachment/anchor points are created from the attachment of three small rectangular layers of polyethylene mesh (Darice Inc., United States) stitched together by stainless steel wire 0.3 mm (Scientific Wire Company, United Kingdom). To generate a suitable anchor 0.5 mm hooped wire was bent over the edge of the side of the well. This meshed wire structure is known as the a-frame. The chamber dimensions are 14 mm × 30 mm × 10 mm and the volumetric capacity is 1.5 mL. Manufactured chambers are fabricated from PEEK, a plastic that is biocompatible for use with cell cultures (Panayotov et al., 2016). The custom manufactured chamber has in-built cylindrical attachment/anchor points, that are posts set within the wells (**Figure 1**). The chamber dimensions are 10 mm × 21.5 mm × 5 mm, and the volumetric capacity is 0.5 mL (**Table 2**). The PEEK chambers are precision machined, with final chamber geometries etched into the plastic, and are based on an outline similar to the CAD design in **Figure 1C** – PEEK. These PEEK chambers were designed to be used within standard 6-well plates. The PEEK chambers were kindly donated by Dr. James Phillips (University College London, United Kingdom).

**Table 2:** Comparison of the chamber features used in this investigation to generate tissue engineered skeletal muscle.

	8-Well chamber	PEEK chamber
**Muscle chamber features**		
Attachment/fixed points	Polyethylene mesh attachment	PEEK pins
Seeding conditions	Both 4 × 10^6^ cells/mL of collagen used	
Construct volume (mL)	1.5	0.5
Geometric configuration	Rectangular	Rectangular
Design type	Custom-built	Commercially available – precision manufactured
Well type	Tissue culture plastic – rectangular 8-well	PEEK wells with posts
Chamber cost	£4.37 per chamber	£99.60 per chamber (autoclavable)


### Cell Seeded Collagen Skeletal Muscle Constructs

Type 1 rat tail collagen hydrogels were polymerized as previously described (Smith et al., 2012). The seeded collagen hydrogels were made to a measure of: 85% v/v type 1 rat tail collagen [2.05 mg/mL; First Link (UK) Ltd., United Kingdom], 10% of 10× minimal essential media (MEM; Gibco, United Kingdom), 5% v/v growth media (GM) containing C2C12s at cellular density of 4 × 10^6^ cells/mL. Prior to the addition of cells, the collagen-MEM solution was neutralized by the drop-wise addition of sodium hydroxide (NaOH; Sigma-Aldrich, United Kingdom) at 5 and 1 M aqueous concentrations. Neutralized acellular collagen solution remained on ice before and after the addition of C2C12s. The homogeneous mixed seeded constructs (0.5 mL for PEEK chambers and 1.5 mL for 8WC model, respectively) were cast into the appropriate chambers and placed in a humidified incubator at 37°C and 5% CO_2_ for 15 min, to allow polymerization. Following polymerization, 6 mL of GM were added to each construct before returning to the incubator. GM was replenished daily, for a period of 4 days, at which point the medium was removed and replaced with differentiation medium (DM) consisting of DMEM supplemented with 2% v/v horse serum (Sigma-Aldrich, United Kingdom) and 1% v/v penicillin–streptomycin (Gibco, United Kingdom). DM was replaced daily for the remaining 10 days of culture. Three replicates were created for each construct and six independent experimental repeats were conducted with a total “*n*” number of constructs being 18 per chamber type; 12 for histochemistry and 6 for gene expression analysis.

### Fluorescent Staining

Following 14 days in culture, the medium was removed from the wells of both configurations and constructs were fixed using 4% paraformaldehyde for a minimum of 60 min. Subsequently, constructs were cut away from the attachment mesh and fixed points and washed three times with 1× Tris-buffered saline (TBS). Constructs were then submersed in 300 μL of 0.2% v/v Triton X-100 (Fisher Scientific, United Kingdom) and diluted in TBS for 120 min. Following three further washes with TBS, constructs were incubated overnight with rhodamine-phalloidin (Life Technologies, United Kingdom) diluted 1:200 v/v in TBS. The following day, constructs were washed three times with TBS prior to incubation with 300 μL of 4′,6-diamidino-2-phenylindole (DAPI; Life Technologies, United Kingdom) diluted 1:2000 v/v in TBS for 10 min. Following a final three washes with TBS, constructs were placed on polylysine-coated microscope slides (VWR, United Kingdom) and mounted to a coverslip using Fluoromount^TM^ (Sigma-Aldrich, United Kingdom) mounting medium.

### Microscopic and Macroscopic Images

**FIGURE 2:**
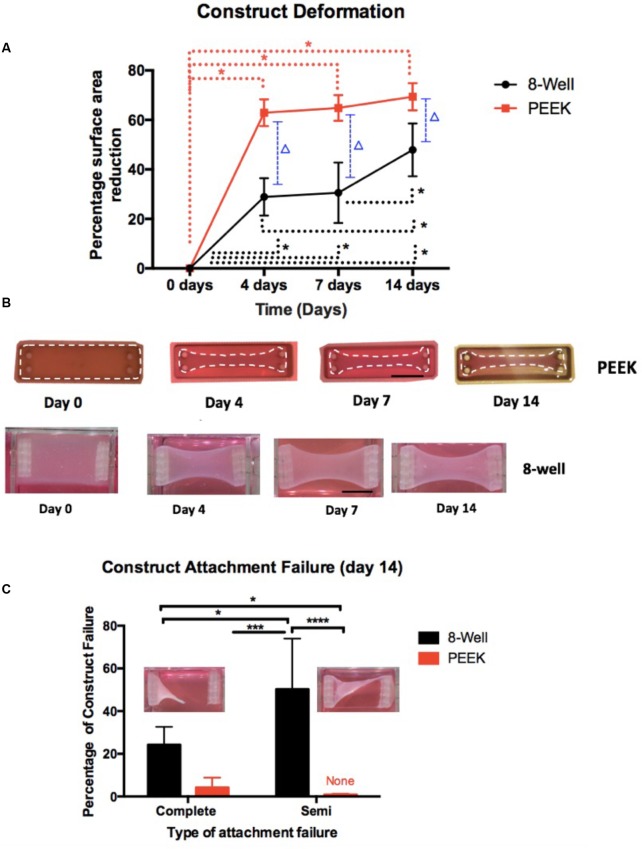
Construct deformation (percentage of construct surface area reduction) over time (a maximum of 14 days) and percentage of attachment failure.**(A)** Construct deformation shows the area of construct reduction over the course of the experiment at key intervals (0, 4, 7, and 14 days). The dotted lines with an asterisk show the link between the time intervals that have significance (*p*-value < 0.05) within the chamber type. The dotted line with a triangle (*p-*value < 0.0001) across the chamber types. **(B)** Macroscopic images of the constructs at days 0, 4, 7, and 14 showing the constructs deformation over the course of the experimental time frame. The upper images are of PEEK constructs that have been enhanced by outlining the constructs to define their contrast within their chamber against the surrounding medium. The lower row images are of the 8WC. Scale bar = 10 mm. **(C)** Attachment failure shows that 8WC has the highest construct failure for both complete and semi-failure, and the image inserts depict attachment failures. For panel **(C)** significance at ^∗^*P* < 0.05, ^∗∗∗^*P* = 0.0002, ^∗∗∗∗^*P* < 0.0001.

Images of fluorescently stained TE SkM constructs were obtained using a confocal microscope (Zeiss LSM 880, Carl Zeiss, Germany) (**Figure 2**). Sets of 60 images were taken of myotubes within the constructs of each chamber type. Macroscopic images of whole constructs within their chambers (to assess macroscopic contraction) were taken throughout the duration of the experiment (**Figure 2B**). Images captured were taken using a digital camera (PEEK only) or a flatbed scanner (Epson V370) (8WC only).

### Image Analysis of Seeded Collagen Skeletal Muscle Construct

All images (micro and macroscopic) were analyzed using FIJI Software by Image J (NIH, Bethesda, MD, United States) to collate the data for the different parameters required for the assessment of the two configurations. The following list of measurements were obtained for each image: myotube width, myotube length, fusion index, number of myotubes, cell density, and the number of nuclei per myotube. Myotubes were classified as elongated structures containing three or more nuclei within a single membrane structure. Irregular mass, clumps, or multi-branched aggregation conformations (complex dysmorphic myotubes) with three or more nuclei were not counted as myotubes. Most myotubes were aligned to the uniaxial isometric lines of strain within the gel, however, some singular branched dysmorphic myotubes were counted. Myotube diameter was calculated as the average of 10 measurements along the myotube length (Rommel et al., 2001; Agley et al., 2012) for a representative measure. The fusion index was calculated as the number of nuclei incorporated into myotubes expressed as a percentage of the total number of nuclei in the image frame (Martin et al., 2015).

### RNA Extraction and RT-qPCR

3D TE SkM constructs for both chamber types were detached from their anchor points and transferred to sterile 1.5 mL microcentrifuge tubes containing 500 μL of TRI Reagent (Sigma-Aldrich, United Kingdom). The homogenization process (maximal shear) was achieved using a needle (23/21G) and syringe technique. RNA extraction was conducted according to the TRI reagent manufacturer’s instructions (Sigma-Aldrich, United Kingdom) using chloroform, 2-propanol and 70% v/v ethanol reagents (grade 200-proof, Sigma-Aldrich, United Kingdom). RNA quality and quantity were measured by a NanoDrop 2000 spectrophotometer (Thermo Fisher Scientific, United Kingdom). Real-time quantitative polymerase chain reactions (RT-qPCRs) were prepared in triplicate in 348-well plates, where each well contained 20 ng of RNA diluted in 5 μL of RNase free water, 0.1 μL of forward and reverse primers (Sigma-Aldrich, United Kingdom; see **Table 3**), 0.1 μL of RT mix (Qiagen, Germany) and 4.7 μL of SYBR green mix (Qiagen, Germany) to make 10 μL total reaction volumes. One-step RT-qPCR was performed on a Viia7^TM^ Real-Time PCR system (Applied Biosystems/Thermo Fisher Scientific, United Kingdom), which was programed to perform the following: 10 min at 50°C (to enable reverse transcription), 5 min at 95°C (to activate “Hot Start” Taq polymerase), followed by 40 cycles of 95°C for 10 s and 60°C for 30 s. Data was analyzed using the comparative C_T_ otherwise known as the Livak method (Schmittgen and Livak, 2008) and relative gene expression 2^(-ΔΔ*C*_T_)^ method using RP2β as the reference gene. The muscle markers selected as primers (**Table 3**) were myogenin (MYOG) an indicator of myogenic differentiation and matrix metalloproteinases (MMPs)-2 and -9 as indicators of matrix remodeling.

**Table 3:** Primer sequences used for detection of differentiation and extra-cellular matrix remodelling mRNA markers.

Gene	Forward primer sequence	Reverse primer sequence	Function
RP2β	GGTCAGAAGGGAACTTGTGGTAT	GCATCATTAAATGGAGTAGCGTC	Housekeeper
Myogenin	CCAACTGAGATTGTCTGTC	GGTGTTAGCCTTATGTGAAT	Differentiation
MMP-2	GAGATCTTCTTCTTCAAGGAC	AATAGACCCAGTACTCATTCC	Matrix remodeling
MMP-9	CTGGCAGAGGCATACTTG	GCCGTAGAGACTGCTTCT	Matrix remodeling


### Statistical Analysis

All data sets are presented as the mean value ± standard deviation per condition on day 14. Normality tests were conducted to evaluate the distribution of the data. *t*-Tests were conducted for myotube analysis tests and (factorial) two-way ANOVA for construct deformation; both tests were used to determine if statistical differences existed between the two different construct chambers. *t*-Tests were conducted using GraphPad Prism software V6 (GraphPad Software Inc., United States). Factorial ANOVA was conducted using IBM SPSS version 23 (International Business Machines Corp., United States). Significance was set at an alpha value of *p* ≤ 0.05.

## Results

### Construct Deformation and Failure Rates

Construct area reduction (deformation) was measured over the experimental duration of 14 days (**Figure 2**). Morphologically, the percentage area of reduction for the 8WC and PEEK constructs increased over time (4 days: 28.89 ± 7.55% 8WC vs. 62.88 ± 5.44% PEEK, *p*-value < 0.0001; 7 days: 30.57 ± 12.17% 8WC vs. 64.86 ± 5.25% PEEK, *p*-value < 0.0001; 14 days: 47.87 ± 10.70% 8WC vs. 69.39 ± 5.50% PEEK, *p*-value < 0.0001). This demonstrates, that mean percentage deformation (reduction in area) is greater in the PEEK than 8WC at all time points analyzed (excluding day 0). Interestingly, the 8WC constructs failed to reach 50% deformation by end the end of the experiment, which may well be a result of differences in construct volume and total cell number compared to the PEEK system. **Figure 2C** highlights the failure rates for both 8WC and PEEK constructs. It was noticed that PEEK constructs only failed within the first 24 h due to failed attachment to the posts. Complete failure (total detachment from anchor posts) and partial detachment was more frequent for the 8WC than for the PEEK, suggesting collagen attachment and remodeling is different between systems. Complete and semi-failure rates, reported significant differences between chamber types (**Figure 2**, semi-failure: 50.21 ± 23.74% 8WC vs. 0% PEEK, *p* < 0.0001, complete failure: 24.2 ± 8.44% 8WC vs. 4.20 ± 4.60% PEEK, *p* = 0.05). This large variability in failure rates highlights the difficulty in reliability and handling of the custom-built chamber and its construct, respectively.

### Morphological Parameters of C2C12 Myotubes Within 3D Tissue-Engineered Constructs in Different Chamber Configurations

**FIGURE 3:**
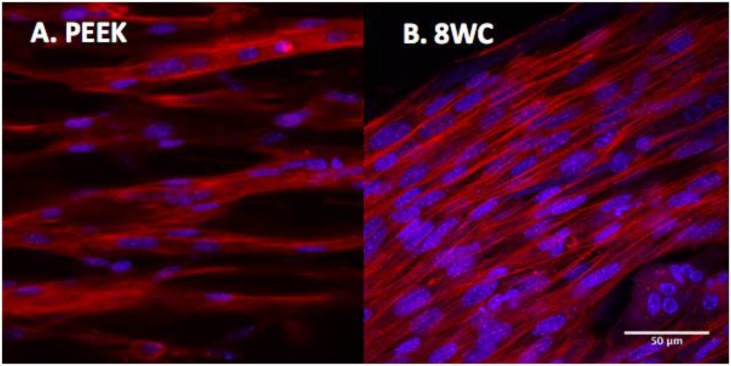
C2C12 myotubes within collagen matrix of 3D tissue-engineered constructs. Confocal images at 14 days. Histochemical staining with rhodamine-phalloidin (actin filaments in red). Cells nuclei are counterstained with 4′,6-diamidino-2-phenylindole (DAPI; nuclei in blue). **(A)** PEEK constructs and **(B)** 8-well constructs. Scale bars at 50 μm.

To determine the overall level of morphological differentiation (and variable differences), myotube parameters (myotube; width, length, number, number of nuclei per myotube, and fusion index) were measured based on fluorescence imaging of the actin cytoskeleton (**Figure 3**). This allowed a detailed comparison between both systems to be made (**Table 4**). Myotube parameters for the 8WC model were found to be generally higher (increases in mean values), compared to the PEEK chamber. However, no statistical differences were noted for any measures (all *p* > 0.05), indicating that the morphological outcomes were reproducible between both chamber types. This suggests that despite differences in volumes, dimensions, and anchor types between chamber types, the degree to which myotubes form at a given seeding density may be limited.

**Table 4:** Parameters used to assess morphological difference after 14 days in culture.

	8-Well chamber	PEEK chamber	*p*-values
**Morphological measures**			
Myotube width (μm)	14.32 ± 2.10	14.00 ± 2.75	0.9949
Myotube length^∗^ (μm)	172.10 ± 18.35	165.9 ± 11.82	0.9672
Number of myotubes (au)	25.00 ± 8.74	21.17 ± 8.23	0.8109
Number of nuclei per myotube (au)	6.818 ± 1.32	8.770 ± 2.03	0.1000
Cell density (au)	139.0 ± 26.45	124.6 ± 28.34	0.6611
Fusion index (%)	45.93 ± 6.94	52.87 ± 2.96	>0.9999
Distance between myotubes (μm)	14.47 ± 5.55	8.63 ± 2.58	0.414
Number of myotube branches (au)	3.17 ± 1.83	2.32 ± 1.47	0.261


*Mean measures of the myotube parameters and their units from their respective constructs (chamber type). Au, arbitrary units. ^∗^Due to limitation of the image frame myotube length measurements are possibly greater than that measured.*

### mRNA Expression Levels for Differentiation and Remodeling Matrix Markers at Day 14

**FIGURE 4:**
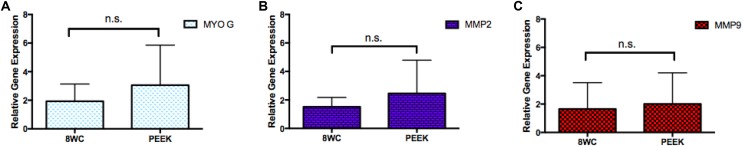
mRNA expression levels for differentiation and remodeling matrix markers. Gene expression at day 14 for **(A)** MYOG, **(B)** MMP-2, and **(C)** MMP-9. No significance difference (n.s.), *p*-values all measured >0.99.

Myogenic gene expression markers are indicators which can be used to gain insight into the progress of these early SkM cells (myoblasts) toward cellular differentiation (Bentzinger et al., 2012). Levels of mRNA expression for differentiation (MYOG) and ECM remodeling markers (MMP-2 and -9), were compared between the PEEK chamber and 8WC (**Figure 4**). There were no significant differences between the two chambers for all genes measured, with similar *p*-values (all *p* > 0.99), supporting the morphological outcomes described above, and a trend toward biological reproducibility within the model irrespective of tissue chamber manufacturing method.

## Discussion

TE of 3D SkM is based on the developmental biology and regeneration of native tissue, i.e., the ability of myogenic precursors to activate, proliferate, and differentiate into multinucleated myotubes in 3D (Okano and Matsuda, 1997, 1998; Okano et al., 1997). Consequently, many SkM models (**Table 1**) display three common features: (i) high cellular density (Khodabukus et al., 2007; Mudera et al., 2010); (ii) the ECM/scaffold used (Bian and Bursac, 2008, 2009); and (iii) the presence of fixed points within the chamber facilitating uniaxial (isometric) tension enabling cellular alignment (**Table 1**). Despite these commonalities, the bespoke nature of these published models means there are many differences in the basic configuration; shape, volume, attachment type, etc.

Here, a comparison between two contrasting systems was made in order to determine differences in myotube parameters. Assessment of fluorescence micrographs of the PEEK chamber vs. 8WC, clearly shows similar myotube morphology, with singular unbranched myotubes regardless of the configuration used (**Table 4**). This response confirms previous publication by this research group, in larger scale models using the similar chamber materials (5 and 3 mL; Sharples et al., 2012; Smith et al., 2012; Player et al., 2014).

Myogenin has been extensively reported to be a late marker of myoblast fusion, required for terminal differentiation (Tan et al., 2015). Mudera et al. (2010) and Smith et al. (2012) demonstrated the expression pattern of MYOG in similar tissue engineered models, where comparisons were made against 2D controls. It was suggested that myotube formation was supported by the extent of MYOG expression (Mudera et al., 2010; Smith et al., 2012), which supports the results reported herein. The expression of MYOG mRNA, showed no difference between configurations tested, suggesting the molecular regulation of differentiation is not altered between conditions.

The similarity in myotube formation also highlights the importance of isometric and uniaxial strain providing ECM cues for directional signaling, independent of anchor type and attachment structure. Cellular attachment, alignment, and ECM remodeling contributes to macroscopic characteristic deformation (Sharples et al., 2012; Smith et al., 2012), causing loss of interstitial fluid (Phillips, 2014). Subsequently, an increase in relative cell density contributes to an increase in cell–cell contact promoting fusion. Despite this, clear differences in construct deformation were found between chamber types. This data contrasts previous observations of ECM remodeling gene expression, where no differences were observed in either MMP-2 or -9 mRNA, which may reflect the post-translational function of these proteins. Indeed, it may well be that within a given configuration (where finite mechanical signals are different) there is a limit in the extent to which matrix remodeling contributes to myotube formation and may also reflect differences at macroscopic and molecular levels.

Attachment failure in both its forms (complete and semi) was more prevalent in the 8WC compared to the PEEK. An overview of the morphological parameters of the constructs from both chamber types, also shows that the PEEK constructs have reduced variability (standard deviation) than 8WC constructs (number of nuclei per myotube, being the exception). Indeed, variability within custom made systems has previously been noted in engineered muscle (Brady et al., 2008; Mudera et al., 2010) and therefore the present results indicate that a precision manufactured PEEK system could lead to improved and more standardized results.

The custom, handmade 8WC produces variation and inconsistencies, both in assembly between researchers and positioning within the chamber. As such, it is likely that this will have a marked effect as to how the constructs attach and develop. By utilizing a commercially available PEEK chamber, consistency and reliability are achieved with this system, particularly as the chambers can be autoclaved and reused. This consistency and reliability becomes more critical when using primary cells types which are difficult to derive, isolate, and culture. Furthermore, this precision manufacturing facilitates the use of automation techniques, which will be a key asset for applications requiring higher-throughput (Vandenburgh, 2010; Nam et al., 2015).

## Conclusion

In this study, 3D TE SkM constructs were fabricated using both commercially available PEEK and custom-built 8WC. Engineered muscle fabricated using PEEK and 8WC were comparable in myotube morphology and mRNA expression, however, the collagen matrix in PEEK constructs deformed and remodeled faster than in the 8WC setup. Importantly, however, the variables measured showed less variability in PEEK configurations compared to 8WC, and displayed dramatically reduced experimental failure rates. The PEEK chamber offers a consistent and reliable system to engineer SkM, however, with no apparent differences in the myotubes that are produced, the resource and particularly the application should be considered when selecting chamber type.

## Author Contributions

JJ conducted the experiments. DP, AC, and NM contributed to experimental work and manuscript editing. VM and ML conducted the final editing and proof reading as well as project supervision.

## Conflict of Interest Statement

The authors declare that the research was conducted in the absence of any commercial or financial relationships that could be construed as a potential conflict of interest.
